# Affects as affordances: disability and the genres of the actionable

**DOI:** 10.3389/fsoc.2024.1410248

**Published:** 2024-12-05

**Authors:** Andries Hiskes

**Affiliations:** The Hague University of Applied Sciences, The Hague, Netherlands

**Keywords:** disability studies, affect, affordance, actionable, genre

## Abstract

Prominent theorists such as Tobin Siebers, Ato Quayson, and Martha Stoddard Holmes have proposed that disability may not only elicit different affects, such as fear, admiration, or disgust, but have also envisioned different ways in which the relationship between affect and disability is becoming a central concern in considering how disability is ultimately lived through and experienced in social life. This paper supplements the conceptualization of the affect–disability relationship with the conceptual apparatuses of affordances and genre, to offer an account of the actionable. The actionable is proposed as a form of socio-cultural negotiation of the body and the environment out of which opportunities for action—or affordances— arise. Thomas Stoffregen has proposed affordances as being relational-emergent in nature, meaning that affordances refer to the possibilities for action within a particular constellation of elements, while simultaneously not being reducible to the properties of the individual elements. This paper proposes that affect, understood as the bodily capacities to act and be acted upon, may be understood as evoking affordances—opportunities to act or be acted upon. Additionally, the notions of impairment and disability suggest that capacities and the possibilities of action may vary across different bodies. I connect this to the work by Lauren Berlant on genre, who suggests that modes of responsivity to being affected are rooted in generic thinking. Genres act as structuring and historical forms that embed affect in appropriate modes of responsivity within genre conventions. Affordances are subsequently linked to what is deemed a fitting action within a genre. By invoking Berlant’s work, this paper proposes that the actionable opportunities afforded by bodies are preemptively inscribed in genre conventions, and that the concept of the actionable enables an analysis of which actions are deemed appropriate within genres. Because impaired and disabled bodies have a variety of capacities, these bodies may therefore also hold the capacity to disrupt generic expectations and therefore further emphasize the normativity of the presupposed appropriateness of actions.

## 1 Introduction

In literary and cultural studies, monographs such as *Disability Aesthetics* (Siebers, 2010), *Aesthetics Nervousness: Disability and the Crisis of Representation* (Quayson, 2007), and *Fictions of Affliction: Physical Disability in Victorian Culture* (Stoddard Holmes, 2009) have argued that the ways in which affects aroused by and through disability are necessarily subject to both representation and politicization: representation, because disability has been featured in literary writing, feature films, TV shows, and many other cultural artifacts; politicization, because the ways in which disabilities, as well as affective responses to them, are represented, are to be understood as political issues.

In this paper, I suggest that affective responses to disabilities might be understood as affordances. This paper builds on previous work (Hiskes, 2019), wherein I posited the concept of *affective affordances*, which concerns the way in which the appearance of, and interaction with, disabled bodies afford affective responses in relation to other bodies. That paper’s primary concern lies with how *reading* for disability concerns the relationship between the form of the bodily impairment and the form of the representation. As affects cannot be represented directly via signs and symbols as Armstrong (2000, p. 124) has argued, the question that paper addresses is how reading for the forms of representations of disability can be generative of affect.

My main concern in the present paper is to delineate how affects may be understood as affordances, which are commonly understood as opportunities for action. I argue that responses to being affected are inscribed within socio-cultural genres, which carry generic conventions as to how a subject *should* act within a specific genre. In other words, that genres have a normative function regarding the appropriateness of actions. However, as not all bodies have the same capacity to act or be affected, I consequently argue that disability holds the potential to disrupt generic conventions of seemingly appropriate actions. The main question this paper explores is consequently how, when a body is affected, it concurrently affords opportunities for action as well as to be acted upon, and how disability often inconveniences the normative generic expectations associated with certain actions. This inconveniencing of disability allows me to theorize what I call the actionable: the socio-cultural negotiation of how a body perceives, is affected by, and acts within an environment, and how we may consequently analyze the appropriateness of actions alongside generic conventions and expectations.

The scholarly literature on the relationship between affect, emotion, and disability remains somewhat limited. Within the existing body of scholarship, several disability theorists have taken an ethico-political approach to the emotion–disability connection. For example, some of Brian Watermeyer’s work argues against the nature of the pervasive connection he sees as being made between loss and disability. Watermeyer (2014, p. 101) explains how, due to disability often being valued as a negative characteristic, the connection between disability and loss remains persistent as a projection. Consequently, he suggests that “loss and other painful aspects of our existence” should be reclaimed (Watermeyer, 2009 p. 100). Similarly, Bill Hughes writes on the relationship between disability and disgust that “Disgust in the presence of disability is a form of cowardice in the face of inevitability and a failure to recognize that mortality is not an enemy but simply the price one pays for life” (Hughes, 2012, p. 73). In arguments like those of Watermeyers and Hughes’, specific affective states like loss or disgust are decoupled from being central to a conception of disability, as these authors argue that affective states such as loss and disgust are in fact pervasive across abled and disabled bodies alike. By persisting on the reiterative cultural connection of loss and disgust with disability, these authors thereby perceive a risk of the enhancement of ableism.

While I have no qualms with the type of arguments Watermeyer and Hughes make, I want to consider such ethico-political approaches to the affect–disability relationship in light of an argument made by Vehmas and Watson (2016), concerning normativity *within* disability studies itself. They note that “Disability studies has always included a strong normative dimension, founded as it is on a belief that life for disabled people could be better coupled with a desire to identify and challenge what are seen as discriminatory practices and beliefs. All theoretical accounts in the field contain either implicit or explicit normative judgments about the ethical or political issues that affect disabled people’s lives” (4). Watson and Vehmas point out how disability studies as a field are intrinsically linked to challenging discriminatory practices toward disability, which consequently leads to it being normative in that it seeks to challenge discriminatory practices and beliefs. Although I agree with Vehmas and Watson’s argument, I also want to take it one step further. Rather than only saying the judgment made by disability studies scholars often have normative content, I also argue that the ethico-political approach *itself* is normative in the sense that it gives primacy to the focus on moral judgments in disability studies, often in seeking to combat or undo ableism.

However, as seen in the examples of Watermeyer’s and Hughes’ work above, such lines of critique tend to forego how disability may or could fundamentally influence a conceptualization of affect and the way it acts upon bodies. In other words, this paper does not seek to supplement the line of ethico-political critiques regarding the connection between certain affective states and disability. Instead, its focus is on the question of how disability may problematize the very notion of affect as what acts upon bodies and causes bodies to act, as disability can effectively question the notion that all bodies are affected the same way or that disabled bodies possess the ability to respond similarly to various affects as non-disabled bodies do.

The motivation for linking the triad of disability–affect–affordance to the notion of genre, is that, as mentioned above, genres carry a set of conventions as to how a subject *should* act, which therefore imbues genre with a normative function. In their book *Cruel Optimism* (Berlant, 2011), Lauren Berlant explores how different kinds of “adjustments to the present” or “the activity of being historical” (20) are grounded in how such activity finds its genre (like narrative, or a soliloquy, or a situation). Ultimately, however, Berlant’s interest, as well as my own, lies in how such adjustments to the present and the activity of being historical are manifested in “explicitly active habits, styles, and modes of responsivity” (20). I argue that affects are not themselves a mode of responsivity, as for a body to be affected by another body, event or object simply means that it is acted upon. Rather, to be affected requires a mode of responsivity or an adjustment to the present.

This negotiation between the way in which a body is affected may translate into an appropriate mode of responsivity is what I designate as *the actionable*. The actionable concerns how opportunities for action, or affordances, may emerge when a body is affected and is required to respond or adjust in some way, which, following the study by Berlant, is seen as necessarily socio-historical. This is consequently linked to the notion of genre as delineated by Berlant, which involves the way in which certain modes of responsivity are deemed to be in line with genre conventions, and thereby considered appropriate. However, as disability problematizes preconceptions concerning what may count as a valid or appropriate action, the actionable in relation to disability can never be understood as a simple *given*. This is why I designate the actionable to be a socio-cultural negotiation, as, though all bodies can and will be affected, not all bodies may have the same modes of responsivity available to them. This negotiation between how a body may translate its being affected into a mode of responsivity can thus allow one to gain and develop further understanding concerning what preconditions are posed on a body to be understood as being ‘able to act’ in a given generic context. Adjacently, and of equal importance, is the fact that there are also many different modes of *in*action that disabled bodies afford and that inactions may disrupt genre conventions. Consequently, affordances are not to be understood here to contain any moral content, such as the notion that a mode of action would be preemptively more desirable than a mode of inaction.

In what follows, I sequentially unpack and delineate the three key terms of this paper—affordance, affect, and genre—and how they relate to disability and to each other. As mentioned in the previous paragraphs, what is ultimately at stake in this paper is examining how disability may inconvenience generic expectations as to how bodies *should* act in relation to how we conceptualize how bodies *can* act. What I have called the actionable thus involves the examination of how and when a body does not act in line with the expectations of a specific genre, which thus, in turn, allows one to query what this means for how we conceptualize ability/disability within that generic context.

## 2 Affordance theory and disability

The term *affordance* was originally coined by social psychologist Gibson (2014), who employed the term to show how affordances constitute a relationship of possibilities for action between two or more elements. As an example, Gibson offers that supportability exists when an extended surface is rigid enough to support the weight of a specific animal (119). In other words, affordances arise out of the meeting of these elements (in this case animal and surface) and the affordances that emerge are particular to that relationship. This conceptualization of affordance is therefore relational-emergent in nature in that it does not define affordances as properties *of* objects, but as relationships that emerge due to the *meeting* of objects (and their accompanying properties). In this conception, I follow Thomas Stoffregen (2003), who has delineated affordances as being relational-emergent in this way (Stoffregen, 2000a, 2000b). This conception of affordance as relational-emergent is distinct from the conception of affordance as was posed by Turvey (1992), who ultimately posits, in Stoffregen’s words, that an affordance “is not a property at the level of the animal–environment system; Turvey was explicit in defining affordances as properties of the environment only” (2003, p. 122). This distinction matters because in Stoffregen’s conceptualization it is not only the properties of the environment that may afford certain opportunities for action, but rather that “the animal–environment system has properties that differ qualitatively from properties of the animal and of the environment; that is, the animal–environment system has emergent properties that do not inhere in properties of the animal or of the environment, considered separately” (Stoffregen, 2003, p. 123).

In Stoffregen’s conceptualization, the *emergent* properties of an animal–environment system (which we may relate to a disability–environment system as well) cannot be reduced to an enumeration of the properties perceived as belonging to the elements themselves. Rather, they are understood as novel properties that emerge as a result of this meeting. This conceptualization is to a degree adjacent to the social model of disability. That model posits that disability arises out of an interaction between a person with an impairment and an environment (both social and material) that disables them (Shakespeare, 2017). Understood through the lens of affordances, the social model might then be understood as a way to consider how environments might offer ‘inaffordances’, i.e., limitations of action. However, if one compares this model to Stoffregen’s definition of affordances, this definition will not hold conceptually, as the properties of the impaired body and the properties of the environment do not account for all the affordances produced by the ‘impairment–environment’ system. Thus, even if an environment may foreclose certain opportunities for action, there may also arise different affordances out of this system. As an example, one might consider how stairs are commonly associated to be walked on, but they might also be crawled on—even if this might not be deemed to be normatively appropriate.

Affordances, according to Gibson (2014, p. 127), are morally neutral in the sense that whatever is considered a positive or negative affordance is always related to the way in which they are perceived by an observer. My contention in this paper is that affects related to, or evoked by, disabled bodies are then also to be considered neutral in the sense that Gibson delineated it. In relation to the social model, the way in which disabilities are generated through the meeting between an impaired body and environment, thereby manifesting perceived blockages of action, may then be perceived to be a kind of negative affordance or inaffordance.

Whereas the social model seeks to importantly stress that impaired bodies become disabled *because* of the way an environment is organized and structured, affordance theory assigns the possibilities of action that emerge to the body–environment system as a whole, which allows for the emphasis on the unicity of affordances that arise out of that system. In her book *Activist Affordances: How Disabled People Improvise More Habitable Worlds* (Dokumaci, 2023), anthropologist Arseli Dokumacı offers an impressive study of the different kinds of affordances that arise out of the often creative ways people with disabilities use their environment. As an example, Dokumacı describes how an elderly man with rheumatoid polyarthritis, Henri, uses the stability of a small dinner table to lean on that table and securely place his coffee mug flat on the table without spilling (4). In effect, it is the quality of the stability of the table that Henri perceives that allows him to figure out a way to place the mug on the table due to his impaired mobility. Such a use of the dinner table—not only using it to place objects on but also to lean one’s body on it for support—is thus a good example of how properties are *emergent* due to the meeting of a particular body and object out of which such opportunities for action may arise, that might not even be perceived as viable or relevant actions by other bodies.

Dokumacı also notes that affordance theory “does not have any way of accounting for actions and behaviors that take place yet correspond to affordances whose possible behaviors or actions require enormous amounts of *effort*, *endurance*, and *ingenuity* to be realized by impaired humans” (51). The emphasis on effort and endurance in the quote suggests that affordances as perceived by people with disabilities are affectively charged. As with Henri’s example, actions cost something of the body and affect them in turn. Interestingly, the relationship between affect and disability is not further delineated in Dokumacı’s study, and it is this relationship to which I now turn.

## 3 Disability studies and the ethico-political approach to affect

As delineated in the introduction of this paper, the scholarly literature that specifically engages with the relationship between affect theory and disability studies are primarily focused on the ways in which certain affective states are repeatedly connected to disability. As mentioned, several articles engage with affects such as loss (Watermeyer, 2009; Watermeyer, 2014) or disgust (Reeve, 2018). An overview article (Goodley et al., 2018) explores how concepts introduced by different affect theorists, including Sara Ahmed (2007, 2010, 2014) and the aforementioned Lauren Berlant (2007) may be relevant in theorizing the relationship between affect theory and disability studies. The article by Goodley et al. therefore seeks to transpose concepts introduced by Ahmed (the feminist killjoy) and Berlant (the notion of ‘slow death’) to disability (by introducing the notion of the ‘crip killjoy’, for example).

The aforementioned Quayson (2007) posits that “Contradictory emotions arise precisely because the disabled are continually located within multiple and contradictory frames of significance within which they, on the one hand, are materially disadvantaged, and on the other, have to cope with the culturally regulated gaze of the normate” (18). According to Quayson, this leads to what he calls aesthetic nervousness, which means that the way in which people with disabilities are interpreted in literary texts is coextensive with the way they are interpreted out of that context (19). Although Quayson does not link his study to affordance theory, the fact that his study links the practice of interpretation to the question of affect (namely that the interpretation of disability is evocative of nervousness) one can posit as affordance in that disabilities *evoke* a mode of action (interpretation) that becomes affectively linked to contradictory emotions. Similarly, Tobin Siebers (2010) has posited that the increase in the representation of disability in modern art needs to be embraced and that “disability enlarges our vision of human variation and difference, and puts forward perspectives that test presuppositions dear to the history of aesthetics” (3).

What these examples have in common is what I have called the ethico-political approach to the affect–disability relationship. Provocatively, the connections made between disability and affect by the theorists above all carry a moral aspect. For Quayson, nervousness is evoked through the activity of interpretation, but this is an ethical query. For Siebers, the increase in disability in modern art is something that should be celebrated as bodily variation. For Goodley et al., the crip killjoy is a figure that is disadvantaged in a society that privileges self-sufficiency. While these connections are all well-argued for, the fact that they immediately link the disability–affect relationship to one with ethics and politics inadvertently bypasses how affects evoked by and through disability may be understood to deepen how we conceptualize both disability *and* affect.

What these authors share is a primary interest in the ways in which disabled bodies affect and are perceived by other bodies, and what certain problematic aspects to that may be in how these affects operate socio-culturally. However, these theories bypass the question of how disability itself may inform a theory of affect, for what body is presumed not only to affect, but also to be affected? As was shown above in my brief exposition of affordance theory, affordances are necessarily matters of perception—that a body, being affected in its environment, comes to recognize opportunities for action that are characteristic to the specific combination of *that* body in *that* environment (as was illustrated with the example of Henri and the table). However, this raises the question about what, if any, the presumptions are about the body that perceives those opportunities for action.

As much work done in disability studies critiques and counters pre-established normative (and often ableist) frameworks, they may unwittingly also set a normative expectation to the way in which affect relates to disability, i.e., that some affective responses might be considered to be more desirable than others. Furthermore, the very question of affective desirability neglects the fact that affect cannot be preemptively responded to or altered into a seemingly more desired response. As I argued above, modes of responsivity are themselves responses *to* affect. Thus, while I do not argue to curtail scholarly discussion concerning the ethical dimensions of affective responses (such as nervousness or the celebration of bodily diversity), this should be separated from the question of whether affective responses themselves can be preemptively (i.e., normatively) deemed to be desirable, to which I answer in the negative, as further explained below.

Through establishing a link between affect and ethics and politics, questions of the affordances of the affects that disability evokes remain largely overlooked. One could link Quayson’s argument that the evocation of nervousness through the interpretation of disability is an affordance of affect. However, Quayson immediately reframes this matter as one that concerns ethics. As I argue that disability may offer insights into how affect itself is conceptualized, I now analyze some definitions of affect in order to propose how theories of disability may influence that conceptualization.

## 4 Between capacities and affordances: impairment’s relation with affect

In *The Ascent of Affect* (Leys, 2017), Ruth Leys traces the different ways in which emotion and affect have been conceptualized across the social sciences and humanities. Referring to the writings of Massumi (2015, 2021), one approach is to define affect as non- or pre-personal forces (distinguishing it from emotional states), which Leys summarizes as “formless, unstructured nonsignifying forces or ‘intensity’” (313). Gregg and Seigworth (2010), who are coming from a materialist perspective, are in line with this definition and define affect as follows:

Affect arises in the midst of inbetweenness: in the capacities to act and be acted upon. Affect is an impingement or extrusion of a momentary or sometimes more sustained state of relation as well as the passage (and the duration of passage) of forces or intensities. That is, affect is found in those intensities that pass body to body (human, nonhuman, part-body, and otherwise), in those resonances that circulate about, between, and sometimes stick to bodies and worlds, and in the very passages or variations between these intensities and resonances themselves. Affect, at its most anthropomorphic, is the name we give to those forces—visceral forces beneath, alongside, or generally other than conscious knowing, vital forces insisting beyond emotion—that can serve to drive us toward movement, toward thought and extension, that can likewise suspend us (as if in neutral) across a barely registering accretion of force-relations, or that can even leave us overwhelmed by the world’s apparent intractability (1).

The definition by Gregg and Seigworth opens by linking affect directly to action. This is because, as the second sentence explains, affect already *acts* upon bodies—it passes from body to body. The second half of the quote again emphasizes action, but this time to explain that affect can *drive* a body toward movement, i.e., action, which, importantly, they signify as ‘a barely registering accretion of force-relations’, meaning that, even if affect can work upon a body, the ability of that body to register the force that acts upon it is not a pre-emptive given, allowing the affected individual to be left overwhelmed. This quote thus offers crucial insight into the different elements that constitute affect: it acts upon bodies; it establishes relationships between different bodies (human or otherwise) through its acting; it can set bodies in motion through being affected; it is not necessarily registered which kind of forces are acting upon the body; i.e., affect may resist processes of identification and registration that can be reductive in nature. Affect can therefore be ‘other than conscious knowing’.

Given the emphasis Seigworth and Gregg put on affect to act upon bodies, this allows me to further elucidate the relationship between affect and affordance. For both Gibson and Stoffregen, affordances are opportunities for action that arise out of the combination of two elements (e.g., a body and an environment), in which that constellation affords specific modes of opportunity for action to arise. Thus, a body that is affected to act within a given environment may then be understood to respond *to* being affected, which is a mode of responsivity in the way that Berlant uses this term, in other words, an adjustment to the present.

As was argued by disability theorists in the treatment of disability theory above, they consistently maintain the need for the recognition of variance and diversity between bodies, which should then also be applied for how bodies can react differently to being affected—in other words, produce different modes of responsivity. This argument is relevant to the way in which we may consider the way affect operates, specifically the bodily ‘capacities to act and act upon’. Here, I want to create a connection between this statement and the cultural model of disability. As sociologist Anne Waldschmidt (2017) observes, the distinction made between impairment and disability allows us to question in what ways impairment itself, referring to the material and physical reality of the body, is mediated through discourse, as disability is socio-culturally constructed through a meeting between an impaired person and a (disabling) environment. Elsewhere, Waldschmidt (2018, p. 75) explains what one of the lines of thinking a cultural model of disability may offer is that “this model understands impairment, disability, and normality as categories generated by academic knowledge, mass media, and everyday discourses. In short, they are “empty signifiers,” which as a concept implies that the signifier (the word) and the signified (the content a word evokes) have a contingent relation and terms do not simply denote reality but constitute the “things” they talk about”. This emphasis on the discursive generation of not just disability, but also bodily impairment and normality, reifies the notion that expectations concerning the way in which bodily capacities should be translated or signified into ‘appropriate’ or ‘normal’ modes of action, are themselves artifacts of culture. Or, as the philosopher Wendell (1996, p. 34) has put it “the distinction between the biological reality of disability and the social construction of a disability cannot be made sharply”. Importantly then, the cultural model allows one to give an account of bodily and lived experience of impairment *in relation* to the social and cultural forces that shape disability (Snyder and Mitchell, 2006).

Thomas (2012, p. 211) has argued in favor of what she calls a materialist ontology of impairment and impairment effects, the latter referring to the way in which impairments influence one’s embodied functioning in the social world, recognizing that both impairments and their effects are socially and culturally constructed. However, Thomas (2014, p. 14) also holds on to the notion that, while recognizing that impairment itself is socio-culturally constructed, “we should not give the bio-medics exclusive rights over the concept of impairment, not perform the poststructuralist ‘vanishing act’ involved in treating *real bodily* var*iations from the average* as entirely linguistically or culturally constructed differences. What is required, I suggest, is a theoretical framework that recognizes *the social dimension of the biological* and the irreducibly *biological dimensions of the social*”. While the present paper does not offer an entire comprehensive framework that Thomas calls for, it does offer a perspective on what I see as the *inherent entanglement* of the social and the biological as a starting point of analysis for the way in which bodily capacities can come to culturally signify as impairments and disabilities, through the (normative) operations and conventions associated with different cultural genres.

Given the cultural understanding and construction of both disability *and* impairment, I argue that the affective capacity to act and to be acted upon, in relation to disability, cannot be thought separately from impairment in the sense that the notion of impairment suggests bodily diversity *in* these capacities referred to. In other words, disability may complicate the definition offered by Gregg and Seigworth by pointing out that such capacities can themselves never be a given but are a variable across bodies. Additionally, how a body in turn *responds* to it being affected, that is, to have a mode of responsivity to affect, is equally variable and may be implicated by impairment. This argument both recognizes the ‘biological reality’ of impairment that Thomas refers to, given the recognition of the diversity of capacity across bodies, yet simultaneously asserts that it is not possible to conceive of ‘impairment’ *without* a socio-cultural context, like genre, in which bodily capacity becomes appraised as impairments in the first place.

What I want to suggest is that the definition of affect as put forth by Gregg and Seigworth offers up many questions that pertain to disability, or formulated more strongly, *should* not be thought of without considering disability. For just as the capacities to act and be acted upon vary between bodies, and may even vary within different bodily states in one body, so too is the question of the ‘registering’ affect in ‘conscious knowing’ not preemptively the same question to all bodies. What I call *the actionable* involves the way in which affordances, conceived of as opportunities for action, necessarily involve the fact that the kinds of opportunities that are perceived as ‘available’ are a negotiation between the capacity for a body to be affected (which varies among bodies), and the way into which this may translate into a mode of responsivity, which is necessarily influenced by the socio-cultural forces referred to by Waldschmidt and Thomas. Consequently, opportunities for action and modes of responsivity are also not free from normative expectations. To elucidate how a body that is affected may determine a suitable mode of responsivity, I turn to the notion of genre, as it can delineate how modes of responsivity, which, following Waldschmidt, are discursively produced through cultural means, get embedded within conventions appropriate to that genre.

## 5 Organized inevitably: thinking of actions in genres

Above, I briefly mentioned how Berlant is interested in the way the activity of being historical relates to how such activity finds its genre. Genre is commonly thought of as involving acts of classification, particularly in relation to literature, film, music, and other art. However, major early theorists of the genre, such as Fowler (1982), already argued that thinking about the genre as a classificatory scheme is limited. Instead, genre can act as a communication system in the sense that once genres are identified they tend to offer a set of expectations and conventions to their audiences (1982, p. 256). Consequently, when one is being confronted with the fact that genre conventions are *not* met, one may point to what one presupposed the convention to be (rather than that what it necessarily is). Berlant’s thinking on genre has been described as a way to give “an account of the relation between affect and the aesthetic” (Cefai, 2023, p. 269). This implies that what Berlant refers to as ‘the activity of being historical’ involves the way in which particular social conduct (which Berlant sees as necessarily cultural-historical) finds its own specific esthetic forms to mediate the appropriate social conduct. Duschinsky and Wilson (2015) have delineated Berlant’s concept of genre as follows:

For Berlant, a “genre” is an emotionally invested, patterned set of expectations about how to act and how to interpret, which organises a relationship between the acting and interpreting subject, their feelings and impressions, their struggles and their historical present. Genres also organise conventions about what might be hoped for, explicitly or secretly, and the bargains that can be made with life. Genres serve as mooring, or placeholders, for intensities within streaming experience. Their conventions give a place and pacing to—and thereby partially hollow out—the discrepancies and the possibilities which occur within the constitution of a particular form of feeling subject (179).

As this quote shows, genre encompasses a myriad of aspects concerning the way in which a subject adjusts to living in their historical present. The ‘emotionally invested, patterned set of expectations about how to act and interpret’ suggests not only that there is a normativity associated with how to act but also that genre implicitly lays a connection between affect and behavioral pattern. In other words, the conventions associated with a genre carry their own affective charge *toward* the expected actions involved. To illustrate this, Berlant (2011, p. 5) offers the example of the situation as a genre which organizes subjects in a particular way: “A situation is a state of things in which something that will perhaps matter is unfolding amid the usual activity of life. It is a state of animated and animating suspension that forces itself on consciousness, that produces a sense of the emergence of something in the present that may become an event”. In a situation, there is a given state of affairs that makes up for one’s everyday life. However, as one recognizes that one is in ‘a situation’ (e.g., a failed relationship and a loss of direction of one’s career), what comes to matter is the sense of the emergence of an event that radically alters the situation *qua* situation, i.e., that radically upends this state of affairs.

Genre consequently organizes affect in a way that is not only associated with that genre’s conventions, but rather, affect is also imbued with the set of *expectations* one carries within the boundaries of a genre, or as mentioned in the quote above “what might be hoped for” (or, just as well, dreaded). Not only does genre therefore organize an affective relationship regarding the way one *should* act or interpret within the confines of a genre (thereby espousing normativity), it also affectively organizes one’s horizon of expectation, originally coined by literary scholar Jauss (1982) to defer to a common set of expectations and anticipations.

Elsewhere, Berlant (2001, p. 46) writes that “For genre to exist as a norm it has first to circulate as a form, which has no ontology, but which is generated by repetitions that subjects learn to read as organized inevitably”. Genre, then, establishes a connection *of* social form (that is, a set of habits and actions deemed appropriate to and expected from that genre’s conventions), but it also carries with it a sense of inevitability, through which genre is imbued with its normative power. In other words, this suggests that not only is genre loaded with expectations through the way subjects read the genres they live through but also it is affectively charged as being predetermined from the outset.

Given this notion of genre as producing a repetitive and reiterative social form of how to act in a given context, I now want to link back to the notion of affordance as shown above. In the example taken from Dokumacı’s study, Henri uses the dinner table in a way that breaks with conventional use; he leans on it to balance himself. In genres that may be commonly associated with the use of dinner tables—the chit-chat, the family dinner, the meeting—their respective affordances do not necessarily endorse the use of tables as objects to secure one’s stability. In fact, they may advise against it. Such non-normative use of the dinner table is a way in which disability disrupts the normative expectations associated with the coffee table and its conventional usages. Simultaneously, this affordance of the usage of the coffee table arises in part because Henri’s mobility is impaired: “he has a very limited range of motion in his wrists, which affects their flexion and extension, Henri described with almost mathematical precision how he puts a full mug on the table without spillage” (2). As such, in the constellation between Henri, his coffee mug, and the coffee table, a beyond-normative affordance of the coffee table can emerge.

If we bring Berlant’s work on genre in relation to the work on affordance, a provocative query can now be offered: how does the relational-emergent notion of affordance relate to Berlant’s conceptualization of genre as providing normative expectations in relation to how we may conceive of the actionable? As Berlant’s argument is that modes of responsivity to being affected are determined by the expectations set by a genre that a subject finds itself in, affordances, as opportunities for action, are relational-emergent *in relation* to genre. In other words, the convention that certain actions would be appropriate to particular genre conventions is something that disability is able to be disrupt and challenge precisely when new affordances arise due to the novelty of how impaired bodies can interact with their environment. Consequently, I argue that disability is crucial in conceptualizing the move from being affected to a mode of responsivity and action, precisely because disability is disjunctive to *both* the capacity to be affected *and* the ability to act.

I can now delineate further why I have called the actionable a matter of socio-cultural negotiation. Affordances arise as properties of the body–environment system as a whole, where a body perceives opportunities to act because it is affected by that environment. This in turn offers a space of negotiation on how to act within that space. Since opportunities for action, as Berlant’s work argues, are inscribed within generic conventions. The negotiation of how to act is not necessarily a process of conscious decision-making, as modes of responsivity appropriate to a genre are not explicated. However, as the example of Henri shows, beyond-normative usage of one’s environment can make us aware of what such genre conventions actually are. Leaning on a dinner table for support may actually be dismissed by others as inappropriate or potentially dangerous use, whereas sitting down for a chit-chat at the same table would not raise any questions.

In their later work, Berlant states in the context of the affective force of inconvenience that “what’s in front of you is not all that’s acting on or in you” (Berlant, 2022, p. 3). In other words, Berlant reminds us that direct perception of one’s environment does not entail the entirety of the ways in which an environment affects the body. However, as I argue, being affected does offer the opportunity to attune the subject to the negotiation concerning how one’s capacities to act and be acted upon may translate into modes of responsivity suitable to the genre one is living through. This attunement may also involve the possibility of the ‘inaffordance’, a foreclosure of action that is relational-emergent to the specifics of that genre. If genre conventions can prescribe appropriateness in relation to actions, this may also allow one to question that appropriateness through the inaffordances that arise.

## 6 Discussion

This paper has explored the intricate relationships between affordance, affect, genre, and disability, arguing for a nuanced understanding of how these concepts interrelate. By examining the relational-emergent nature of affordances, this paper highlights how opportunities for action arise not from the properties of the environment or the body, but that properties are emergent from the meeting between the two as a system. Affect, understood as the capacities of bodies to act and be acted upon, plays a crucial role in this dynamic, influencing how affordances are perceived and can be enacted by impaired and disabled bodies.

Genres, as socio-historical constructs, embed normative expectations about appropriate actions and modes of responsivity. Lauren Berlant’s work on genre illuminates how these expectations shape and are shaped by affective responses, structuring the ways bodies are perceived and how they are expected to act. This paper has posited that disabled bodies, by their very nature, challenge and disrupt these normative expectations.

The actionable, as proposed in this paper, represents the socio-cultural negotiation of how bodies perceive, are affected by, and act within their environments and can consequently comply with or resist generic conventions. This concept is pivotal in understanding how the socio-cultural mediation of impairment, as has been argued by proponents of the cultural model of disability, may take place. I argue that recognizing these dynamics is essential for the possibility of a more comprehensive socio-cultural analysis of the relationship between disability and action to take place and what is perceived and/or sensed as being *valid* actions.

As this paper has sought to argue that disability may complicate and enrich the relationship between affect and affordance, the question that I would like to close with is the question that may arise whether disability offers its particular own modes of responsivity, or whether the argument could even be made that disability may produce its own genres. Certainly, disability is a staple trope in what is called ‘genre fiction’—which refers to demarcated literary genres such as horror, fantasy, and romance. In her book *Disability, Literature, Genre*: *Representation and Affect in Contemporary Fiction*, Cheyne (2019) examines the relationship between these different genre fiction and disability. She concludes that, while genre *can* resist or even adjust ableist representations of disability, it can also reproduce or encourage disabling attitudes (166).

One way in which Berlant (2018) delineates the complication of how genre pervades normativity in both its affective horizon of expectation and those habits and behaviors it deems appropriate to generic conventions is through the concept of so-called *genre flailing*:

Genre flailing is a mode of crisis management that arises after an object, or object world, becomes disturbed in a way that intrudes on one’s confidence about how to move in it. We genre flail so that we do not fall through the cracks of heightened affective noise into despair, suicide, or psychosis. We improvise like crazy, where “like crazy” is a little too non-metaphorical (2018, p. 157).

For Berlant, genre flailing happens due to the instability and uncertainty of how to move in one’s disturbed object world. In other words, genre flailing occurs at the moment when a subject is confronted with an event where the normative conventions associated with that genre do not work, and there arises a need for continuous recalibration to that object world (the type of activity Berlant refers to as crisis management). This quote establishes a link between disruptive and erratic behavior and how such behaviors may discombobulate genre conventions. It is not my intention here to argue that people with disabilities may be considered experts in crisis management due to the often unstable object worlds that they venture and live in. As I have shown with my delineation of the actionable, this involves a theory of how the possibility of action may arise in an environment but might also cause friction with the appropriateness of action. Genre flailing, then, can be understood as both intruding on one’s confidence in navigating their object world while simultaneously undermining the nature of generic convention.

The cultural model of disability makes a distinction between impairment and disability, where the claim is that impairment, too, is socially and culturally mediated. A theory of the actionable, or how opportunities to act may even arise, I believe is important in further understanding how such processes of mediation can operate culturally. Genre flailing, which Berlant describes as ‘a little too non-metaphorical’, thus points to the nature of the body that is perceived as acting outside of generic conventions, as disabled bodies are often perceived as doing. This importantly links the category of action to that of culture; i.e., it suggests that the non-metaphorical nature of flailing that Berlant refers to may also point to bodies that are perceived as acting ‘out of control’ in specific generic contexts.

When bodies do not function in a way that is in line with generic conventions, Berlant points out that falling ‘through the cracks of heightened affective noise’ leads subjects into bodily states where the issue of control over the body is exactly the issue that comes to be at stake. The terms Berlant gravitates toward to describe subjects overwhelmed by such affective noise—despair, psychotic, crazy—all refer to states in which impairment becomes an inconvenience not only with regard to not fitting in with genre expectations but rather disrupts the presupposed affordances associated with that genre, i.e., the set of opportunities for action as defined by a genre’s horizon of expectation. Consequently, when impairment becomes inscribed as a disability within a genre, the notion of ‘capacities to act and be acted upon’ is always present to simultaneously hold the capacity to disrupt that genre, but also, incidentally, to attune people to what the genre’s conventions were—it may attune subjects to those very conventions. Berlant wrote that inconveniences make you aware of the fact that ‘what’s in front of you is not all that’s acting on or in you’. Impairments, then, can consequently heighten us to the cultural conventions of genres we live through.
